# The Effects of Synthetic SREBP-1 and PPAR-γ Decoy Oligodeoxynucleotide on Acne-like Disease In Vivo and In Vitro via Lipogenic Regulation

**DOI:** 10.3390/biom12121858

**Published:** 2022-12-12

**Authors:** Hyemin Gu, Hyun-Jin An, Mi-Gyeong Gwon, Seongjae Bae, Christos C. Zouboulis, Kwan-Kyu Park

**Affiliations:** 1 Department of Pathology, School of Medicine, Catholic University of Daegu, Daegu 42472, Republic of Korea; 2 Departments of Dermatology, Venereology, Allergology and Immunology, Dessau Medical Center, Brandenburg Medical School Theodor Fontane and Faculty of Health Sciences Brandenburg, Auenweg 38, 06847 Dessau, Germany

**Keywords:** decoy oligodeoxynucleotide, lipogenic regulation, peroxisome proliferator activated receptor-γ, sterol regulatory element-binding protein-1

## Abstract

Acne vulgaris has a pathogenesis that involves increased sebum production and perifollicular inflammation. Sterol regulatory element-binding protein-1 (SREBP-1) and peroxisome proliferator activated receptor-γ (PPAR-γ) are transcription factors that regulate numerous genes involved in lipid biosynthesis. To improve a new therapeutic approach, we designed the SREBP/PPAR decoy oligodeoxynucleotide (ODN), a synthetic short DNA containing complementary sequences for the SREBP and PPAR transcription factors. We aim to investigate the beneficial functions and the molecular mechanisms of the synthetic SREBP/PPAR decoy ODN in lipogenic models. *C. acnes* was intradermally injected with a 1.0 × 10^7^ colony forming unit/20 μL. The synthetic SREBP/PPAR decoy ODN or scrambled decoy ODN (10 μg) was transferred via the mouse tail vein injection. SZ95 cells were transfected with 2 μg of synthetic ODNs. After transfection, the SZ95 cells were cultured in serum-free medium containing 20 ng/μL of insulin-like growth factor-1 (IGF)-1 for 24 h. To investigate the expression of gene and signaling pathways, we performed Western blotting. The distribution of the chimeric decoy ODN was confirmed by EMSA. Lipid levels were assessed by Nile red and Oil Red O staining. The cytokine levels were measured by ELISA kit. This study showed that *C. acnes*-injected mice and IGF-1-stimulated SZ95 cells exhibited increased expression of SREBP-1 and PPAR-γ compared to the normal controls. In contrast, the administration of the SREBP/PPAR chimeric decoy ODN significantly suppressed the upregulation of lipogenic genes. Furthermore, the SREBP/PPAR decoy ODN decreased the plasma cytokines and cytokine levels of total protein. These results suggested that the SREBP/PPAR decoy ODN exerts its anti-lipogenic effects by regulating lipid metabolism and by inhibiting lipogenesis through the inactivation of the SREBP and PPAR pathways. Therefore, the synthetic SREBP/PPAR ODN demonstrates substantial therapeutic feasibility for the treatment of acne vulgaris.

## 1. Introduction

Acne vulgaris, the most common chronic inflammatory skin disease, affects more than 85% of adolescents and adults, and it may leave permanent scars if not treated properly [1]. The important role of increased sebum production in the pathogenesis of acne has long been well established [2]. A growing body of evidence underlines the role of increased insulin-like growth factor-1 (IGF-1) signaling in the pathogenesis of acne, and IGF-I levels showed a positive correlation with the severity of acne in women with clinical acne [3].

IGF-1 is a polypeptide hormone with molecular properties that are very similar to those of insulin, and it plays an important role in regulating cell proliferation and differentiation [4]. IGF-1 stimulates the lipogenic transcription factors peroxisome proliferator activated receptor-γ (PPAR-γ) and sterol regulatory element-binding protein-1 (SREBP-1), which in turn play fundamental roles in lipogenesis [5]. PPAR-γ and SREBP-1 preferentially regulate the genes involved in fatty acid synthesis, and these lipid metabolic changes favor the overgrowth of *Cutibacterium acnes* (*C. acnes*) and biofilm formation, promote subsequent inflammation, and interfere with follicular barrier function [6].

Sebaceous lipids are regulated by PPARs, a family of nuclear receptors that regulate epidermal growth and differentiation and lipid metabolism [7]. Activated PPARs are well-known ligand-activated transcription factors that regulate the expression of multiple genes involved in the regulation of lipids, glucose, and amino acid metabolism [7]. In particular, PPAR-γ, which is abundantly expressed in the skin, is an important molecule in acne vulgaris as the most frequent sebaceous gland-related skin disease characterized by abnormal lipid accumulation and inflammation [8].

SREBP-1 binds to a sterol response element, a nucleotide sequence found in the promoter regions of several lipogenic genes in the cholesterol and fatty acid biosynthetic pathway [9]. This binding to DNA increases the transcription of target genes and induces sebum fatty acid monounsaturation, which plays an important role in the comedogenesis and inflammation of acne [6,10]. SREBP-1 regulates many target genes, including acetyl-CoA carboxylase (ACC) [11], fatty acid synthase (FAS) [12], stearoyl-CoA desaturase 1 (SCD-1) [13], and HMG-CoA reductase (HMGCR). Therefore, the regulation of the SREBP-1/PPAR-γ signaling may serve as a useful therapeutic tool for treating acne vulgaris.

Currently, a number of skin disorders with an inflammatory component are treated with steroids and/or antibiotics in both oral and topical formulations [14,15]; they are administered to suppress inflammation or kill bacteria. However, long-term use of these drugs has potential side effects such as teratogenicity, dyslipidemia, liver enzyme abnormalities, and development of antibiotic resistance [14,16,17]. Therefore, the need for research on new anti-acne agents is increasing, and more systematic and safe agents are needed. The synthetic decoy oligodeoxynucleotide (ODN) technique is a gene therapy strategy that uses a synthetic double-stranded ODN containing a consensus binding sequence for transcription factors to block the activity of specific transcription factors [18]. Synthetic decoy ODNs have been used to successfully regulate the activity of related genes and proteins, wherein they bind to specific transcription factors and limit the interaction between these transcription factors and their associated binding sites corresponding to gene promoter regions [18,19]. Although these decoy ODNs have been proven to be beneficial in several disease models, whether SREBP-1 and PPAR-γ decoy ODN could attenuate the development of the molecular mechanisms of lipogenesis, which are the major causes of acne vulgaris, has not yet been demonstrated. We therefore attempted to alleviate skin diseases such as acne by regulating lipid synthesis at the transcriptional level by regulating the transcription factors SREBP-1 and PPAR-γ.

Therefore, this study aimed to investigate the beneficial function and efficiency of a therapeutic strategy based on a synthetic SREBP/PPAR decoy ODN in the simultaneous regulation of the transcription factors SREBP-1 and PPAR-γ in *C. acnes*- and IGF-1-induced acne vulgaris models. The synthetic SREBP/PPAR decoy ODN was designed to contain both the SREBP-1 and PPAR-γ binding sequences to effectively block lipogenesis. Considering that acne vulgaris is closely related to lipogenesis, inflammation, and abnormal bacterial growth, this study hypothesizes that a chimeric decoy ODN that is capable of blocking lipogenic transcription factors may be beneficial in the treatment of acne.

## 2. Materials and Methods

### 2.1. Preparation of Bacteria

*C. acnes* (ATCC 6919; American Type Culture Collection, Rockville, MD, USA) was cultured in Brain Heart Infusion Broth (BD, Sparks, MD, USA) under anaerobic conditions using a BD GasPack™ at 37 °C until OD600 = 1.0 (logarithmic growth phase).

### 2.2. Synthesis of Decoy ODN

Considering the feasibility of the decoy ODN strategy, we designed a ring-type synthetic chimeric decoy ODN for SREBP-1 and PPAR-γ consensus sequences. The S-Fold program was used to select the target site for SREBP/PPAR through a sequential overlap simulation of the secondary structure. Synthetic decoy ODNs were synthesized on the Macrogen (Seoul, Korea). SREBP/PPAR chimeric decoy ODN and Scr ODN sequences were utilized (Table 1; the consensus sequences are underlined). These ODN structures were annealed for 6 h while the temperature was gradually decreased from 80 °C to 25 °C. To obtain covalent ligation for the ring-type ODN molecules, we mixed each ODN with T4 DNA ligase (1 U; Takara Bio, Otsu, Japan), and then we incubated them for 18 h at 16 °C.

### 2.3. Animal Models and Transfection of Decoy ODN

The mice were randomly divided into seven groups (six mice/group): normal control (NC), normal control treated with SREBP/PPAR chimeric decoy ODN (S/P), *C. acnes* injection (CA), *C. acnes* treated with scrambled decoy ODN (Scr), *C. acnes* treated with SREBP decoy ODN (CA+S), *C. acnes* treated with PPAR decoy ODN (CA+P), and *C. acnes* treated with SREBP/PPAR chimeric decoy ODN (CA+S/P). Female ICR mice (seven weeks old; Samtako, Daejeon, South Korea) were housed in a room with controlled humidity and temperature and subjected to a 12-h light-dark cycle. After one week of acclimatization, the mice in the synthetic SREBP/PPAR chimeric decoy ODN groups received 10 μg of SREBP/PPAR chimeric decoy ODN via tail vein injection. A nonviral vector Trans IT In Vivo Gene Delivery System (Mirus, Madison, WI, USA) was used to deliver the chimeric decoy ODN. After two days, the mice were put under brief isoflurane anesthesia, and *C. acnes* was intradermally injected (1.0 × 10^7^ colony forming unit/20 μL in phosphate-buffered saline) into both the left and right ears. The NC group mice received 20 μL of phosphate-buffered saline (PBS) alone. After 24 h, a second 10 μg dose of synthetic decoy ODN was administered via tail vein injection. The animals were anesthetized with isoflurane and euthanized 24 h after the second tail vein injection. At the end of each treatment period, blood was collected by cardiac puncture from the mice, and the mice were euthanized by CO2 asphyxiation. Subsequently, their ear skins were excised for the next experiments. Fluorescein isothiocyanate (FITC)-labeled ODN was injected using a Label IT nucleic acid labeling kit (Mirus Bio, Madison, WI, USA) to examine the transfection efficiency of the synthetic ODN. Ear skin samples were embedded using an optimum cutting temperature compound (Sakura Finetek Japan, Tokyo, Japan), and then frozen sectioning was performed. Animal care and all experimental procedures were approved and conducted in accordance with the guidelines of the Institutional Animal Care and Use Committee of the Catholic University of Daegu (Approval Number: DCIAFCR-190626-09-Y).

### 2.4. Cell Culture and Treatment

SZ95 cells, the immortalized human sebaceous gland cell line [20], were cultured in Dulbecco’s Modified Eagle Medium (DMEM)/F-12 supplemented with Glutamax ITM, 10% fetal bovine serum, 100 U/mL penicillin, 100 μg/mL streptomycin (Gibco, Grand Island, NY, USA), 1 mM CaCl_2_, and 5 ng/mL human epidermal growth factor (Sigma-Aldrich, St. Louis, MO, USA) at 37 °C in a 5% CO_2_ humidified incubator. Cells were sub-cultured every 3–4 days at approximately 85% confluence.

The SZ95 cells were seeded in complete medium for 24 h. Then, the medium was replaced with fresh serum-free medium. The SZ95 cells were transfected with 2 μg of synthetic ODNs for 6 h using Lipofectamine 2000 (Invitrogen, Carlsbad, CA, USA) according to the manufacturer’s instructions. After transfection, the SZ95 cells were cultured in a serum-free medium containing 20 ng/mL of IGF-1 for 24 h. The SZ95 cells were divided into five groups: normal control (NC), normal control treated with SREBP/PPAR chimeric decoy ODN (S/P), IGF-1 stimulation (IGF-1), IGF-1 treated with Scr decoy ODN (IGF-1+Scr), and IGF-1 treated with SREBP/PPAR chimeric decoy ODN (IGF-1+S/P).

### 2.5. Histological Analysis

All skin tissue specimens were fixed in 10% formalin for 24 h at room temperature. They was dissected, dehydrated, and embedded in paraffin. Then, thin sections (4 μm) were mounted on glass slides and stained with the hematoxylin and eosin (H&E). As part of the histological assessment, all slides were examined using a Pannoramic^®^ MIDI slide scanner (3DHISTECH). Redness was analyzed using the i-Solution DT software (IMT i-Solution) based on the entire image taken at the same location, location, and angle.

### 2.6. Immunohistochemistry

The paraffin-embedded tissue sections were deparaffinized with xylene and dehydrated in gradually decreasing concentrations of ethanol. For immunohistochemical analysis, the dehydrated tissue sections were immersed in 10 mM sodium citrate buffer (pH 6.0) for 5 min at 95 °C. The last step was repeated using 10 mM fresh sodium citrate solution. The sections were allowed to cool in the same solution for 20 min and then rinsed with PBS. Next, the sections were incubated with a primary antibody for 1 h at 37 °C. The primary antibodies were anti-PPAR-γ (Cell Signaling Technology, Danvers, MA, USA), anti-SREBP-1 (Abcam, Cambridge, Cambridgeshire, UK), anti-FAS (Cell Signaling Technology, Danver, MA, USA), and anti-ACC (Cell Signaling Technology). After three rounds of serial washing with PBS, the sections were processed with an indirect immunoperoxidase technique using a commercial EnVision System kit (DAKO, Carpinteria, CA, USA). The slides were examined with a Pannoramic^®^ MIDI slide scanner, and integrated optical density was analyzed using the i-Solution DT software (IMT i-Solution).

### 2.7. Immunofluorescence Staining

Immunofluorescence staining was performed as previously described [16]. The primary antibodies used were PPAR- γ (Cell Signaling Technology) and SREBP-1 (Santa Cruz Biotechnology, Santa Cruz, CA, USA). The secondary antibodies used were Alexa Fluor 488 and/or Alexa Fluor 594.

The SZ95 cells were fixed with 10% formalin for 15 min at room temperature. The cells were subsequently treated with 0.1% Triton X-100 in PBS for 5 min to permeabilize them. Then, the cells were blocked at room temperature for 30 min and incubated with primary antibody against SREBP-1 and PPAR-γ at 37 °C for 1 h. For double staining, a reaction with Alexa Fluor 488 phalloidin (Thermo Fisher Scientific, Waltham, MA, USA) was performed at room temperature for 1 h. The nuclei were stained with 4′,6-diamidino-2-phenylindole (DAPI) for 5 min at 37 °C. The slides were mounted using VECTASHIELD Mounting Medium (VECTOR Laboratories, Burlingame, CA, USA).

### 2.8. Western Blot Analysis

Total protein samples were extracted from the ear skin and from the cultured SZ95 cells with a lysis buffer (Cell Lytic™ M; Sigma-Aldrich) according to the manufacturer’s instructions. Protein concentrations were determined using a Bradford assay (Bio-Rad Laboratories, Hercules, CA, USA), with absorbance measured at 595 nm using a spectrophotometer. Protein samples were separated using precast gradient polyacrylamide gels (Bolt™ 4–12% Bis-Tris Plus Gels; Thermo Fisher Scientific, Waltham, MA, USA) according to the manufacturer’s recommendations, and transferred to nitrocellulose membranes (GE Healthcare, Chicago, IL, USA). The membranes were blocked using 5% bovine serum albumin and probed with primary antibodies and horseradish peroxidase-conjugated secondary antibody. After the wash step was repeated, the membranes were immersed in enhanced chemiluminescence detection reagents (Thermo Fisher Scientific) for 1 min. Signal intensity was measured with an image analyzer (ChemiDoc™ XRS+; Bio-Rad Laboratories) and quantified using the Image Lab software (Bio-Rad Laboratories) GAPDH (glyceraldehyde-3-phosphate dehydrogenase). The protein expression values were normalized to GAPDH expression values. The primary antibodies used were anti-ACC, anti-FAS, anti-GAPDH (Cell Signaling Technology), anti-SREBP-1, -PPAR-γ, -SCD-1, -interleukin (IL)-1β, -COX-2, -iNOS (Santa Cruz Biotechnology), -CD36, -HMGCR, -tumor necrosis factor (TNF)-α, -interferon (IFN)-γ, and -IL-6 (Abcam). The protein expression values were normalized to GAPDH expression values.

### 2.9. Enzyme-Linked Immunosorbent Assay (ELISA)

A mouse blood sample was collected, and the serum was separated. Serum TNF-α, IL-1β, and IFN-γ levels were measured using Quantikine mouse ELISA kits (R&D Systems) according to the manufacturer’s instructions. Absorbance at 450 nm was determined using an ELISA reader (BMG Labtech, Ortenaukreis, Germany).

### 2.10. Electrophoretic Mobility Shift Assay (EMSA)

Nuclear extract fractionation from cells was performed using an NE-PER Nuclear and Cytoplasmic Extraction Kit (Thermo Fisher Scientific) according to the manufacturer’s instructions. The Lightshift^®^ Chemiluminescent EMSA Kit (Thermo Fisher Scientific) was used for the EMSA to analyze the expression levels of SREBP and PPAR. ODNs consisting of the consensus SREBP-binding site (forward: 5′-AGTCATCACCCCACTA-3′; reverse: 5′-TAGTGGGGTGATGACT-3′) and/or the PPAR-binding site (forward: 5′-CAAAACTAGGTCAAAGGTCA-3′; reverse: 5′-TGACCTTTGACCTAGTTTTG-3′) were used as primers.

### 2.11. Flow Cytometric Analysis

The decoy ODN was labeled with FITC using a Label IT nucleic acid labeling kit (Mirus Bio). SZ95 cells were cultured in six-well plates and transfected with 2 μg of FITC-labeled decoy ODN or with non-labeled decoy ODN using Lipofectamine 2000. For the flow cytometric analysis, the cells were washed, trypsinized, dispersed, and then transferred into 500 μL PBS. The samples were analyzed with CytExpert (Beckman Coulter, Brea, CA, USA).

### 2.12. Nile Red Staining

We diluted a stock solution of Nile Red (Sigma-Aldrich; 1 mg·mL^−1^ in acetone) to a final concentration of 10 μg/mL in PBS. Cells were fixed in 4% formaldehyde at room temperature for 10 min, stained with Nile Red solution for 15 min at 37 °C, and washed with PBS.

### 2.13. Oil Red O Staining

The SZ95 cells were washed with PBS and fixed in 10% formalin for 10 min. The cells were washed and stained with 0.6% (*w*/*v*) Oil Red O (Sigma-Aldrich) staining solution (isopropanol:distilled water in a *v*/*v* ratio of 6:4) for 1 h, washed with distilled water, and then counterstained with hematoxylin. The cells were mounted using a VECTASHIELD Mounting Medium (VECTOR Laboratories) and were examined under a Pannoramic^®^ MIDI slide scanner (3DHISTECH).

### 2.14. Data and Statistical Analysis

All data are presented as mean ± standard error of the mean (SEM). Statistical analysis was performed using GraphPad Prism 5 (GraphPad Software, Inc., San Diego, CA, USA) and SPSS Statistics (IBM, Armonk, NY, USA). Group means were compared by one-way ANOVA with Tukey’s multiple comparison test. Tukey’s tests were run only when F achieved *p* < 0.05 and when there was no significant variance inhomogeneity. Dunnett’s post-hoc test was performed under conditions that did not assume equal variances. Differences with *p* < 0.05 were considered significant. 

## 3. Results

### 3.1. Construction of the Decoy ODNs

**Figure 1:**
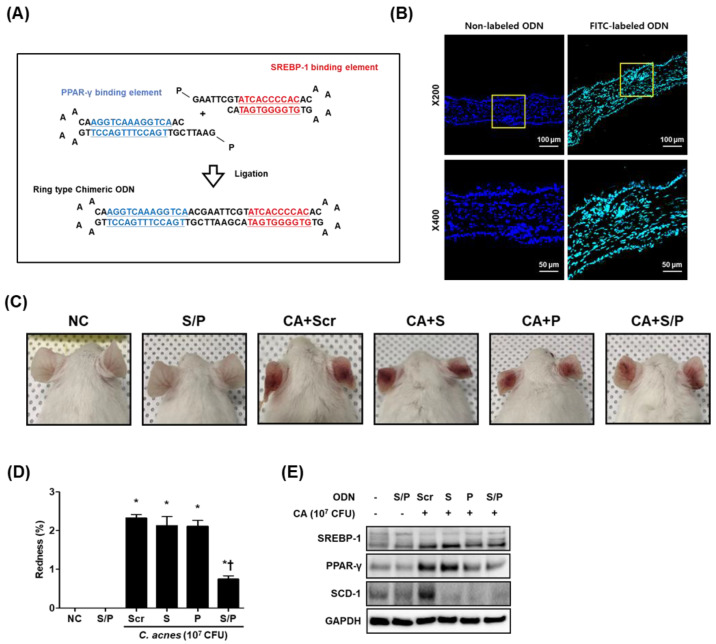
The SREBP/PPAR chimeric decoy ODN was effectively transferred in ear skin. (**A**) Structure of the synthetic SREBP/PPAR chimeric decoy ODN. (**B**) The transfer effectiveness of the SREBP/PPAR chimeric decoy ODN in ear skin (green). DAPI (blue)-stained nuclei. (**C**) Representative mouse ear skin lesion images. (**D**) The redness of the ear skin. (**E**) Single and chimeric decoy ODNs suppressed the protein expression of SREBP-1, PPAR-γ, and SCD-1. * *p* < 0.05 compared with the NC. † *p* < 0.05 compared with the CA+Scr.

The synthetic SREBP/PPAR chimeric ODN was designed to contain the consensus sequences of the SREBP and PPAR transcription factors (Figure 1A). The synthesized double-stranded ring-type SREBP/PPAR chimeric decoy ODN contained the SRE (5′-ATCACCCCAC-3′) and PPRE (5′-AGGTCAAAGGTCA-3′) consensus sequences. As shown in Figure 1B, the FITC-labeled chimeric decoy ODN was detected both in the cytoplasm and nucleus of cells in ear skin. 

### 3.2. The SREBP/PPAR Chimeric Decoy ODN Alleviated C. acnes-Mediated Skin Lesions in the Mouse Model

**Figure 2:**
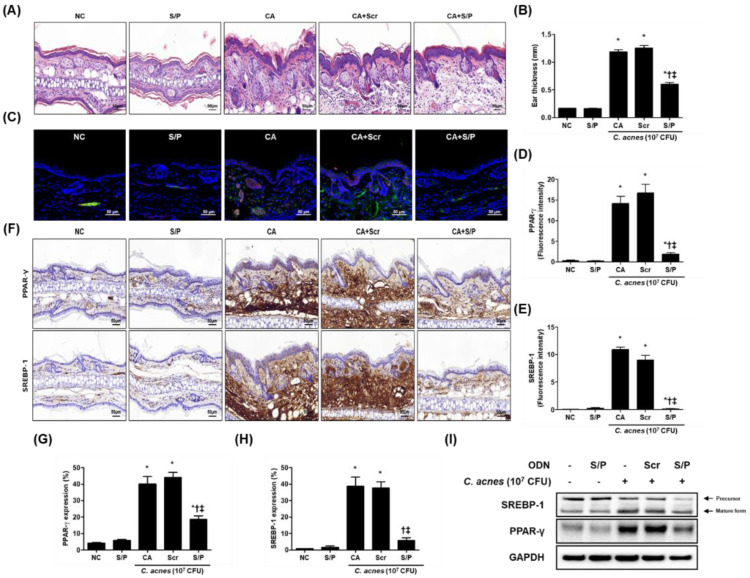
The SREBP/PPAR chimeric decoy ODN reduced the inflammatory infiltration and activities of the lipogenic transcription factors. (**A**) Images for histopathological inflammation severity through H&E staining. (**B**) Ear thickness. (**C**) Immunofluorescence double staining images of (**D**) SREBP-1 (Alexa Fluor 555, red) and (**E**) PPAR-γ (Alexa Fluor 488, green). DAPI (blue)-stained nuclei. (**F**) Immunohistochemical staining was used to evaluate the extent of PPAR-γ and SREBP-1 expression, which was subsequently quantified (**G**,**H**). (**I**) The protein levels for SREBP-1 and PPAR-γ by Western blot analysis. * *p* < 0.05 compared with the NC. † *p* < 0.05 compared with the CA. ‡ *p* < 0.05 compared with the CA+Scr.

The mice injected with *C. acnes* began to show signs of inflammation, such as erythema, which continued to increase in severity until the end of the experiment; by contrast, no symptoms were observed in the mice injected with PBS alone. Interestingly, the mice treated with the synthetic SREBP/PPAR chimeric decoy ODN showed less erythema than the mice treated with *C. acnes* (Figure 1C,D). As shown in Figure 1E, the expression levels of PPAR-γ and SREBP-1 were reduced in the CA+S/P group compared with those in the CA+Scr group. Even lower expression levels of these factors were observed in the group transfected with the chimeric decoy ODN than in the group that received single decoy ODN. H&E-stained skin showed increased ear thickness and infiltration of inflammatory cells in the dermis in the *C. acnes* treatment group. By contrast, the synthetic SREBP/PPAR ODN-treated mice showed a significant reduction in ear thickness and reduced infiltration of inflammatory cells compared with the *C. acnes*-treated mice (Figure 2A,B). 

### 3.3. The SREBP/PPAR Chimeric Decoy Suppressed the Lipogenic Transcription Factors in the C. acnes-Mediated Skin Lesions

The effects of the SREBP/PPAR chimeric decoy ODN on skin lipogenesis were investigated in a *C. acnes*-induced lipogenesis model. The expression levels of SREBP-1 (red) and PPAR-γ (green) increased in the sebaceous glands of mice with *C. acnes*-induced skin lesions compared with those in the normal skin (Figure 2C). As shown in Figure 2C–H, the CA and the CA+Scr group showed higher expression levels of these lipogenic transcription factors in the ear skin compared to the NC group. The administration of the SREBP/PPAR chimeric decoy ODN downregulated the lipogenic transcription factors in the CA+S/P group. This expression trend, as determined using immunohistochemistry, was confirmed by Western blot analysis (Figure 2I). Based on these results, the SREBP/PPAR chimeric decoy ODN possibly protected the skin during *C. acnes* stimulation through the regulation of lipogenesis.

### 3.4. The SREBP/PPAR Chimeric Decoy Attenuated the Expression of Lipogenesis-Related Genes in the C. acnes -Mediatd Skin Lesions 

**Figure 3:**
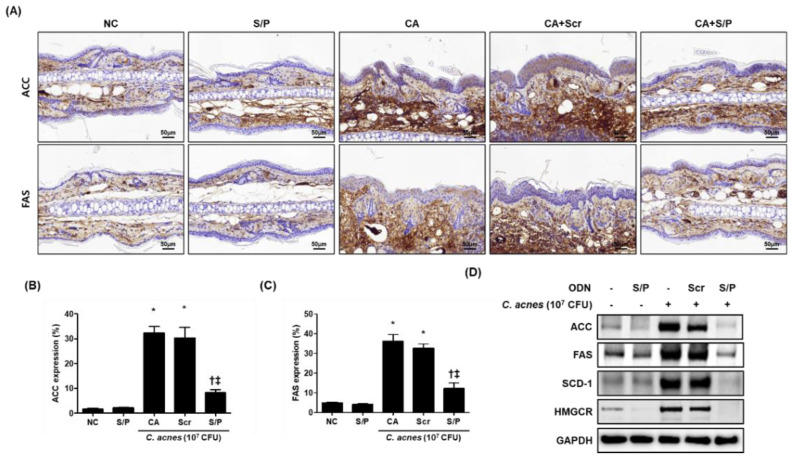
The SREBP/PPAR chimeric decoy ODN significantly inhibited fatty acid metabolism in the *C. acnes*-induced acne lesion mouse model. Representative images obtained from each group. (**A**) Immunohistochemical staining results showing that SREBP/PPAR chimeric decoy ODN inhibited the expression of ACC and FAS, the levels of which were subsequently quantified (**B**,**C**). (**D**) Western blot analysis results showing that the SREBP/PPAR chimeric decoy ODN inhibited the expression of the lipogenic factors. * *p* < 0.05 compared with the NC. † *p* < 0.05 compared with the CA. ‡ *p* < 0.05 compared with the CA+Scr.

To identify the anti-lipogenic effect of the SREBP/PPAR chimeric decoy ODN in acne-related lipid accumulation, we measured the expression levels of ACC and FAS using immunohistochemical staining. The injection of Scr ODN had no effects on the decrease of ACC and FAS caused by *C. acnes*. However, treatment with the SREBP/PPAR ODN reduced the ACC and FAS expression in *C. acnes*-induced skin lesions in mice (Figure 3A–C). Moreover, Western blotting results showed that the SREBP/PPAR chimeric decoy ODN decreased the protein levels of SREBP-1 and PPAR-γ, as well as the key downstream targets of all SREBPs, such as ACC, FAS, SCD-1, and HMG-CoA synthase (Figure 3D). These findings suggested that the SREBP/PPAR chimeric decoy ODN suppressed the expression of lipogenic factors. Taken together, the data indicated that the SREBP/PPAR chimeric decoy ODN suppressed the lipogenic changes and lipid accumulation in the *C. acnes*-injected mice.

### 3.5. The SREBP/PPAR Chimeric Decoy ODN Reduced the Inflammatory Changes in the Animal Model of C. acnes-Like Lesions

**Figure 4:**
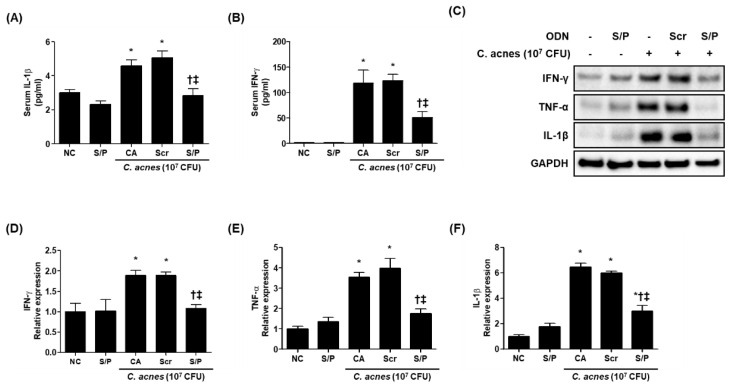
The SREBP/PPAR chimeric decoy ODN suppressed *C. acnes*-induced activation of pro-inflammatory cytokines. ELISA results demonstrating that the SREBP/PPAR decoy ODN suppressed the *C. acnes*-induced increased serum levels of (**A**) IL-1β and (**B**) IFN-γ. (**C**) Representative Western blot analysis results showing that the SREBP/PPAR decoy ODN inhibited the *C. acnes*-induced activation of pro-inflammatory cytokines. (**D**–**F**) Graphs showing the quantitative signal intensities of IFN-γ, TNF-α, and IL-1β after normalization with GAPDH. The results are expressed as mean ± SEM. * *p* < 0.05 compared with the NC. † *p* < 0.05 compared with the CA. ‡ *p* < 0.05 compared with the CA+Scr. Scale bar = 50 μm. NC, normal control; S/P, normal control treated with SREBP/PPAR chimeric decoy ODN; CA, group injected with living *C. acnes*; CA+Scr, group injected with living *C. acnes* and treated with Scr decoy ODN; CA+S/P, group injected with living *C. acnes* and treated with SREBP/PPAR chimeric decoy ODN.

The enzyme-linked immunosorbent assay (ELISA) results showed that the animals treated with the SREBP/PPAR chimeric decoy ODN had significantly reduced inflammatory cytokine levels compared with the Scr decoy ODN-treated group and the CA group. In particular, the serum IL-1β and IFN-γ levels were decreased by the SREBP/PPAR chimeric decoy ODN treatment compared with the Scr decoy ODN treatment, and the SREBP/PPAR chimeric decoy ODN effectively attenuated the production of IL-1β and IFN-γ (Figure 4A,B). The effect of the SREBP/PPAR chimeric decoy ODN on inflammation was also examined by Western blot analysis. As shown in Figure 4C–F, the skin obtained from the NC group showed low expression levels of inflammatory cytokines, such as IFN-γ, TNF-α, and IL-1β. However, the expression levels of IFN-γ, TNF-α, and IL-1β increased in *C. acnes*- and Scr decoy ODN-treated mice, but they were decreased by the SREBP/PPAR decoy ODN treatment. This expression trend based on the Western blot analysis results was confirmed statistically (Figure 4D–F). Treatment with the SREBP/PPAR chimeric decoy ODN statistically decreased the TNF-α and IL-1β levels. These results suggest that the SREBP/PPAR decoy ODN is a potentially effective gene therapy for the prevention of lipogenesis and amelioration of inflammation in acne-like lesions. 

### 3.6. Effects of the SREBP/PPAR Chimeric Decoy ODN on the DNA-Binding Activity of SREBP and PPAR In Vitro

**Figure 5:**
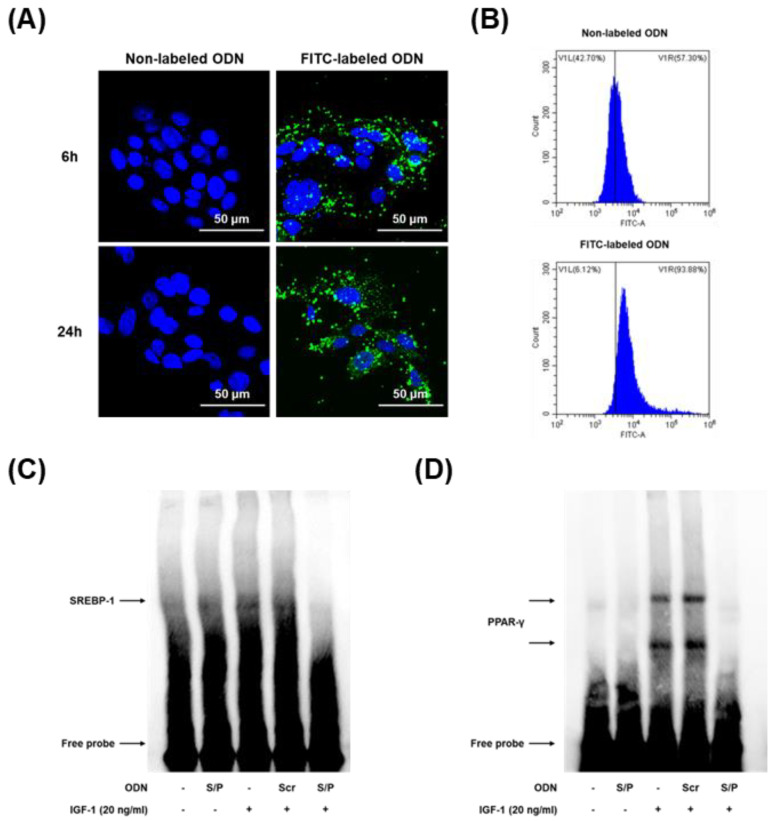
Confirmation of the FITC-labeled SREBP/PPAR chimeric decoy ODN in SZ95 cells. (**A**) Representative immunofluorescence images showing the fluorescence activity in both the cytoplasm and nucleus detected via FITC-labeled ODN deposition (green). DAPI (blue)-stained nuclei. (**B**) The transfection efficiency of the FITC-labeled decoy ODN was measured using flow cytometry. The effect of the SREBP/PPAR chimeric decoy ODN on (**C**) SREBP-1 and (**D**) PPAR-γ transcription activity.

To confirm the efficacy and distribution of the chimeric decoy ODN, we transfected SZ95 cells with FITC-labeled chimeric decoy ODN. The FITC-labeled ODN was successfully transfected, as shown in Figure 5A,B; the synthetic ODN was transferred into the nucleus and cytosol of the SZ95 cells and retained in the cells for 24 h. 

EMSA was performed to analyze the effect of the SREBP/PPAR chimeric decoy ODN on SREBP-1 and PPAR-γ expression levels in IGF-1-induced lipogenesis. SREBP-1 and PPAR-γ binding activities were both significantly increased in the IGF-1 and Scr groups. By contrast, the administration of the SREBP/PPAR chimeric decoy ODN significantly suppressed the activation of SREBP-1 and PPAR-γ in the IGF-1-induced lipogenesis model. Therefore, these findings demonstrated that the SREBP/PPAR chimeric decoy ODN effectively reduced the DNA binding activity of SREBP-1 and PPAR-γ at the transcriptional level in the IGF-1-induced lipogenesis in vitro (Figure 5C,D).

### 3.7. The SREBP/PPAR Chimeric Decoy ODN Decreased the Lipogenic Transcription Factors in IGF-1-Stimulated Human SZ95 Sebocytes

**Figure 6:**
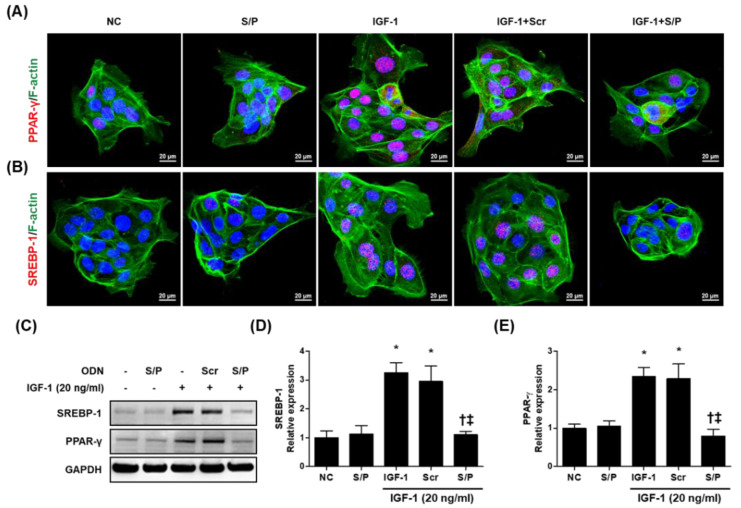
The SREBP/PPAR chimeric decoy ODN influenced the expression of PPAR-γ and SREBP-1 in IGF-1-stimulated SZ95 cells. Representative immunofluorescence images of (**A**) SREBP-1 and (**B**) PPAR-γ (labeled with Alexa Fluor 555, red). F-actin was labeled with Alexa Fluor 488 (green). DAPI (blue)-stained nuclei. (**C**) Representative Western blot analysis results showing that the SREBP/PPAR chimeric decoy ODN inhibited the IGF-1-induced PPAR-γ and SREBP-1 expression. (**D**,**E**) Graphs showing the quantitative signal intensities of SREBP-1 and PPAR-γ. * *p* < 0.05 compared with the NC. † *p* < 0.05 compared with the IGF-1. ‡ *p* < 0.05 compared with the IGF-1+Scr.

To explore the influence of the SREBP/PPAR chimeric decoy ODN on SREBP-1 and PPAR-γ activity, we examined the expression of SREBP-1 and PPAR-γ in SZ95 cells using immunofluorescence staining. The expression levels of SREBP-1 and PPAR-γ in the IGF and IGF + Scr ODN group were significantly higher than those in the NC and S/P decoy ODN group. However, the IGF-1 + S/P decoy ODN treatment group restored the increase to a near-NC level (Figure 6A,B). Similarly, the protein expression of SREBP-1 and PPAR-γ was increased in IGF-1 and IGF-1 + Scr decoy ODN group, which was effectively inhibited in IGF-1 + S/P group (Figure 6C–E).

### 3.8. The SREBP/PPAR Chimeric Decoy ODN Impaired the Intracellular Lipid Accumulation in IGF-1-Treated SZ95 Cells

**Figure 7:**
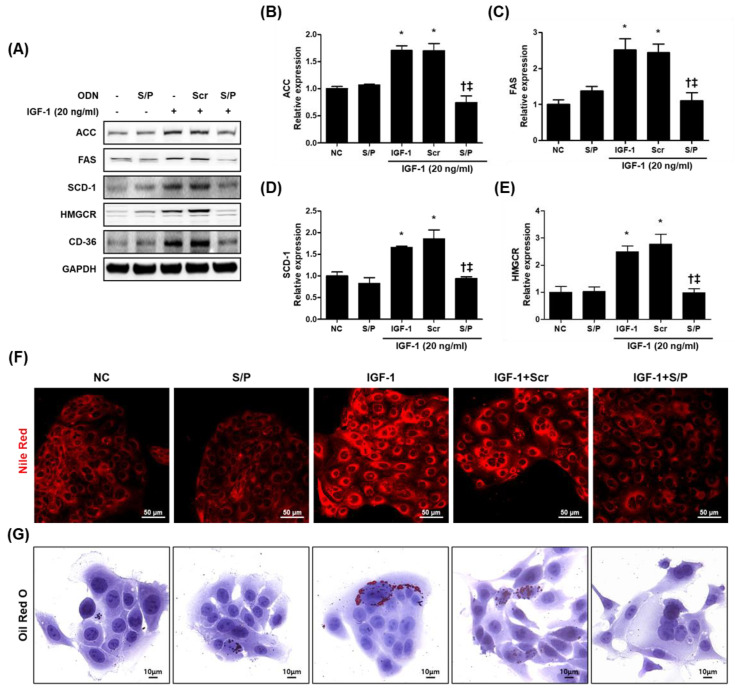
The SREBP/PPAR chimeric decoy ODN significantly inhibited the intracellular lipids in IGF-1-stimulated SZ95 cells. (**A**) Western blot analysis results showing that the SREBP decoy ODN inhibited the expression of ACC, FAS, SCD-1, HMGCR, and CD-36. Expression levels of (**B**) ACC, (**C**) FAS, (**D**) SCD-1, and (**E**) HMGCR. Representative images of (**F**) Nile Red and (**G**) Oil Red O. * *p* < 0.05 compared with the NC. † *p* < 0.05 compared with the IGF-1. ‡ *p* < 0.05 compared with the IGF-1+Scr.

We further investigated whether the blocking of SREBP-1 and PPAR-γ affected the intracellular lipid accumulation induced by IGF-1. As shown in Figure 7A–E, the expression levels of the target genes (i.e., ACC, FAS, SCD-1, HMGCR, and CD-36) of PPAR-γ and SREBP-1 were effectively increased by the IGF-1 treatment compared with the NC and S/P group. However, the expression levels of the target genes of PPAR-γ and SREBP-1 were effectively reduced by the SREBP/PPAR chimeric decoy ODN treatment compared with the IGF-1 and Scr decoy ODN treatments.

Intracellular lipid and lipid droplet formation were investigated using Nile Red and Oli Red O staining. Lipid formation, seen by the Nile Red and Oil Red O staining was increased by the IGF-1 treatment compared with the NC and S/P group. However, the expression levels were reduced by the SREBP/PPAR chimeric decoy ODN treatment compared with the IGF-1 and Scr decoy ODN treatments (Figure 7F,G).

### 3.9. The SREBP/PPAR Chimeric Decoy ODN Suppressed the IGF-1-Induced Inflammatory Responses

**Figure 8:**
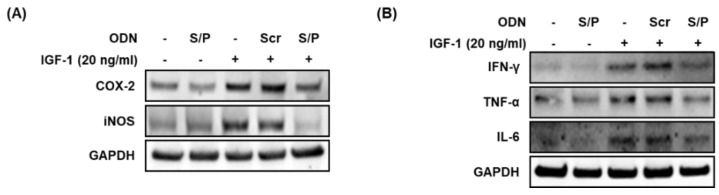
The SREBP/PPAR chimeric decoy ODN suppressed the activation of inflammatory factors in IGF-1-induced lipogenesis. (**A**,**B**) Western blot analysis results demonstrated that the SREBP/PPAR chimeric decoy ODN inhibited the expression of COX-2, iNOS, IFN-γ, TNF-α, and IL-6 in IGF-1-stimulated SZ95 cells.

This study attempted to determine whether an ODN could inhibit the expression and activity of inflammatory factors in sebocytes stimulated by IGF-1. As shown in Figure 7B and Figure 8A, the expression levels of the inflammatory factors were higher in the IGF-1 and Scr ODN groups than in the NC, where the expression levels were rather low. Inflammatory cytokine expression was attenuated by the SREBP/PPAR chimeric decoy ODN treatment compared with the IGF-1 and Scr decoy ODN treatments. The SREBP/PPAR decoy ODN prevented the expression of inflammatory factors involved in IGF-1-induced lipogenesis. Taken together, the data indicate that the SREBP/PPAR chimeric decoy ODN suppressed the expression of proinflammatory cytokines in IGF-1-induced lipogenesis.

## 4. Discussion

Acne vulgaris is caused by the development of sebaceous glands, which are skin appendages that secrete lipids [21]. The proliferation of sebaceous gland cells and the production of sebum are regulated by a complex hormonal system and a variety of factors such as heredity, environmental and metabolic conditions, stress, diet, and injury [22]. Among others, IGF-1 and IGF-1R are well-known targets of *C. acnes* [23], where IGF-1 induces lipid synthesis in human sebocytes and plays an important role in the development of acne [4,5]. Zouboulis et al. [24] have reported increased serum IGF-1 levels in adult women and men with acne, as well as an increased number of total acne lesions and inflammatory lesions. Patients with IGF-1 deficiency, also known as Laron syndrome, do not develop acne or other mTORC1-driven diseases of civilization [25,26]. Several studies have demonstrated that IGF-1 activates the pathways of SREBP-1 and PPAR-γ, leading to a downstream increase in adipogenesis in sebaceous gland cells [9,27]. 

SREBPs are a family of transcription factors that regulate lipid homeostasis and metabolism by regulating the expression of endogenous cholesterol, fatty acids, triacylglycerol, and enzymes required for phospholipid synthesis [9]. The SREBP family consists of three subtypes: SREBP-1a, SREBP-1c, and SREBP-2 [18]. SREBP-1 enhances the transcription of the genes required for fatty acid synthesis. SREBP-2 is primarily responsible for the transcription of cholesterol-related genes, such as the gene for the restriction enzyme for cholesterol synthesis, and the low-density lipoprotein receptor gene [18]. Although a functional overlap exists between SREBP-1 and SREBP-2, SREBP-1 generally controls genes in the fatty acid biosynthesis pathway, whereas SREBP-2 controls the transcription of genes involved in cholesterol biosynthesis [9]. Given that SREBPs play a critical role in lipid synthesis, the inhibition of SREBPs may be a valuable strategy for treating obesity, dyslipidemia, and acne vulgaris.

PPARs act as transcriptional regulators of various genes, such as those involved in lipid synthesis in several organs, including the skin. The three PPAR isoforms, PPAR-α, -β/δ, and -γ, share significant sequence and structural homology, but regulate distinct sets of genes due to differing tissue distribution, selectivity, and reactivity to ligands [28,29]. Among other isoforms, PPAR-α and PPAR-γ are considered the most involved in sebocyte biology [30]. PPAR-α appears to be associated with the β-oxidation and lipid catabolism of fatty acids. PPAR-γ is an essential factor for sebaceous cell differentiation, and it has been reported that sebocytes apparently do not develop from cell lineages devoid of PPAR-γ [31]. Guo et al. [32] have reported that PPAR-γ agonists induce lipid droplet-forming colonies in sebocyte differentiation models. Given that PPAR-γ plays an important role in lipid synthesis, the inhibition of PPAR-γ may also be an important strategy in regulating sebum lipogenesis, sebaceous cell differentiation, and sebum production. 

In previous studies, we have demonstrated that the activation of SREBP-1 and PPAR-γ was accompanied by the increased transcription of several lipogenic genes, the lipogenic genes ACC, FAS, SCD-1, and p70s6k, which are target genes involved in fatty acid biosynthesis [16]. ACC, the major regulator of sebum formation, is mainly involved in the synthesis of long-chain fatty acids and regulates fatty acid oxidation [33,34]. FAS, encoded by the FASN gene, is a multienzyme protein that catalyzes fatty acid synthesis [35]. SCD-1, a gene encoding the key enzyme for the synthesis of monounsaturated fatty acids, is highly expressed in the sebaceous gland [36]. HMGCR is a rate-limiting enzyme in cholesterol biosynthesis, so its activity plays an important role in controlling the synthesis of de novo cholesterol [18]. Therefore, this study investigated the therapeutic effect of synthetic SREBP/PPAR decoy ODN on inflammation and lipogenesis in vivo and in vitro.

Lu et al. [37]. showed that lipogenesis was inhibited through downregulation of the SREBP-1 pathway in human sebaceous gland cells. Zhang et al. [38] demonstrated that the reduction of the expression of SREBP-1 and lipogenic factors, such as ACC, FAS, and SCD-1, improves acne vulgaris. Furthermore, Gu et al. [16]. demonstrated that downregulation of SREBP-1 and PPAR-γ suppressed lipogenesis-specific gene expression. An et al. [18]. demonstrated the efficacy of the SREBP decoy ODN in a hyperlipidemia mouse model. It was confirmed that SREBP decoy ODN reduced total cholesterol and triglyceride levels in the serum of hyperlipidemia mouse models, and also significantly inhibited the expression of inflammatory cytokines. Whether the expression of these major lipogenic factors is regulated is one of the main indicators to determine the inhibitory effect of pharmacologically active substances on lipogenesis. It has been suggested that agents related to the inactivation of SREBP-1 and PPAR-γ could be developed as anti-acne agents with sebum-suppressing effects. Therefore, the specific SREBP-1 and PPAR-γ antagonists could be considered candidates for anti-acne agents.

The present results showed that the activities of SREBP-1 and PPAR-γ were increased in living *C. acnes*- and IGF-1-induced acne vulgaris models. By contrast, the SREBP/PPAR chimeric decoy ODN markedly reversed the responses induced by living *C. acnes* and IGF-1 by suppressing the lipogenic factors through the modulation of SREBP-1 and PPAR-γ. In addition, our study showed that the expression levels of inflammatory cytokines and COX-2 were increased by IGF-1 treatment, whereas SREBP/PPAR chimeric decoy ODN treatment reduced the inflammatory response accompanied with the inhibition of lipogenesis in vitro and in vivo. Although suppressing sebum production is the main therapeutic objective, reducing the level of pro-inflammatory fatty acids can make an important contribution to improving acne. 

Thus, effective treatment that targets the causes of acne vulgaris requires an understanding of the initial molecular mechanisms. Understanding the signaling mechanisms that regulate the production of sebum lipids can help identify potential therapeutic target sites that can reduce sebum production and improve acne. The SREBP/PPAR chimeric decoy ODN exerts its anti-lipogenesis effects in *C. acnes*- and/or IGF-1-induced lipogenesis models by regulating lipid metabolism through the inactivation of the SREBP-1 and PPAR-γ pathways. From this perspective, the SREBP/PPAR chimeric decoy ODN may be an effective agent for the treatment of excessive lipogenesis, such as acne vulgaris.

## 5. Conclusions

This study aimed to understand the mechanism by which SREBP/PPAR chimeric decoy ODN regulates skin lipid metabolism. This study showed that *C. acnes*-injected mice and IGF-1-stimulated SZ95 cells exhibited increased expression levels of SREBP-1 and PPAR-γ compared with the normal controls. In contrast, the administration of the SREBP/PPAR chimeric decoy ODN significantly suppressed the upregulation of lipogenic genes. Furthermore, the levels of lipogenic factors and plasma cytokines in *C. acnes*-injected mice and the cytokine levels of total protein in IGF-1-stimulated SZ95 cells were reduced. These results demonstrated that the SREBP-1 and PPAR-γ pathways independently and cooperatively contributed to acne lesions and that the SREBP/PPAR chimeric decoy ODN reversed the metabolic abnormalities by regulating the SREBP-1 and PPAR-γ signaling pathways. Thus, the results suggested that the SREBP/PPAR chimeric decoy ODN could potentially serve as an additional therapeutic intervention for lipogenesis-related acne.

## Figures and Tables

**Table 1:** ODN sequences utilized in this study.

Oligodeoxynucleotide	Sequence
SREBP-1	5′-GAATTCGTATCACCCCACACAAAAGTGTGGGGTGATAC-3′
PPAR-γ	5′-GAATTCGTTGACCTTTGACCTTGAAAACAAGGTCAAAGGTCAAC-3′
Scramble	5′-GAATTCAATTCAGGGTACGGCAAAAAATTGCCGTACCCTGAATT-3′

## Data Availability

Not applicable.
